# Attix free‐air chamber correction factors computed using EGSnrc

**DOI:** 10.1002/mp.17629

**Published:** 2025-01-25

**Authors:** John T. Stasko, Wesley S. Culberson

**Affiliations:** ^1^ Department of Medical Physics School of Medicine and Public Health University of Wisconsin‐Madison Madison Wisconsin USA

**Keywords:** air kerma, correction factors, free‐air chamber

## Abstract

**Background:**

A cylindrical free‐air chamber, the Attix FAC, is used for absolute air‐kerma measurements of low‐energy photon beams at the University of Wisconsin Medical Radiation Research Center. Correction factors for air‐kerma measurements of specific beams were determined in the 1990s. In order to measure air‐kerma rates of beams in development, new correction factors must be computed.

**Purpose:**

We aimed to compute monoenergetic correction factors for air‐kerma measurements with the Attix FAC that could be used to determine corrections for arbitrary polyenergetic beams.

**Methods:**

A model of the Attix FAC was created in the Monte Carlo code, EGSnrc. The EGSnrc user codes, egs_fac, and egs_chamber, were utilized to calculate aperture transmission, scatter, collecting rod electron loss, and wall electron loss correction factors for incident monoenergetic photon beams with energies between 5 and 50 keV. Beam‐specific correction factors were then derived from the monoenergetic correction factors and compared with the currently accepted values.

**Results:**

Correction factors were computed in 0.5 keV intervals. The newly calculated beam‐specific correction factors and the old conventional values agreed within 0.1% for all beams investigated.

**Conclusions:**

The process for determining monoenergetic correction factors for air‐kerma measurements with a free‐air chamber is detailed in this work. Beam‐specific correction factors can then be calculated if photon spectra are known. This process can be carried out for any free‐air chamber, given specific materials and dimensions for modeling.

## 1 INTRODUCTION

The University of Wisconsin Medical Radiation Research Center (UWMRRC) has developed a free‐air ionization chamber (FAC) to determine the air‐kerma rates of low‐energy x‐ray beams (<50 keV). The name, free‐air chamber, comes from the chamber's lack of a front or rear wall for the external radiation beam to pass through.^1^ The UWMRRC FAC is used as a research instrument to determine air‐kerma rates and should not be confused with the methods used for calibrations at the University of Wisconsin Accredited Dosimetry Calibration Laboratory (UWADCL), which are based on NIST‐calibrated ionization chambers. Just like a conventional ionization chamber, the FAC will collect liberated charge within the central air cavity when irradiated by an external beam. The FAC is designed such that the volume, and subsequently, mass, of irradiated air from which charge is collected within the chamber is well‐known. Researchers then calculate the exposure rate of the beam, which can be used to determine the air‐kerma rate of the beam at the point of measurement (POM).

**FIGURE 1:**
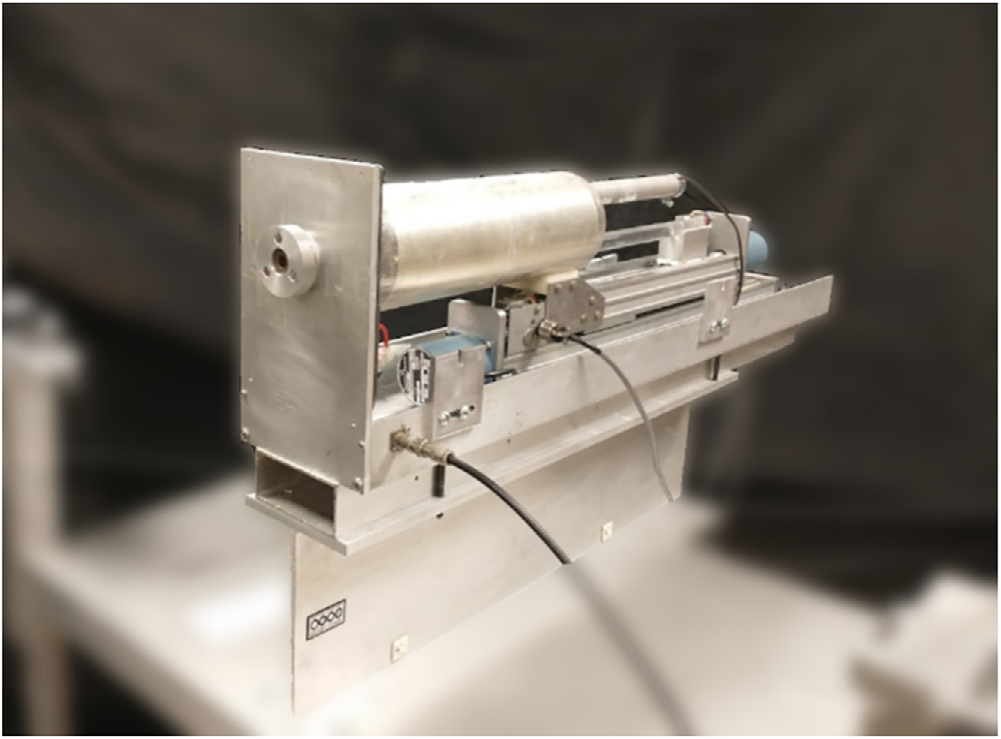
Photograph of the Attix FAC. The x‐ray beam enters through the circular aperture on the left and exits the rear of the chamber. During irradiations, a steel shell is placed around the chamber for electrical safety and to prevent photons from scattering into the chamber from outside the primary collimated beam. FAC, free‐air ionization chamber.

One issue with FACs is that the volume of air from which charge is collected is not always clear and may be difficult to determine. This is due to electric field nonuniformities within the chamber, especially near chamber boundaries. The low‐energy FAC in use at the UWMRRC has a telescoping variable‐length design that was originally proposed by Attix.^2^ A picture of this specific FAC, named the Attix FAC, is shown in Figure 1.

The variable‐length design allows the user to collect two measurements with different collecting volume lengths. The difference between the two measurements is the charge collected from the central region of the larger collecting volume, where the electric field is well‐known and uniform. This avoids the issue of field nonuniformity that is present in the front and rear regions of the chamber.

Several correction factors must be determined for the measured current to accurately correspond to the air‐kerma rate at the POM. Equation (1) presents these corrections and the relationship between the measured current and the air‐kerma rate:

(1) $$\overset{.}{\underset{air}{K}}=\frac{\Delta I}{\Delta L\rho A_{0}}\;{\left(\frac{\bar{W}}{e}\right)}_{air}\frac{1}{1-\bar{g}}k_{att}k_{ap}k_{s}k_{wl}k_{cl}k_{ii}k_{w}$$

where Δ*I* is the change in measured current for a change in the collecting volume length of Δ*L*, *ρ* is the density of air in the chamber, *A_0_* is the area of the chamber's aperture, ${\left(\frac{\bar{W}}{e}\right)}_{air}$ is the average energy necessary for an electron to generate one ion pair in air, and *ḡ* is the average fraction of electron energy lost to bremsstrahlung production.^3^ The various correction factors account for attenuation between the aperture and center of the chamber (*k_at_*_t_), transmission through the chamber aperture (*k_ap_*), the charge generated by secondary electrons from scattered or bremsstrahlung photons (*k_s_*), charge lost due to electrons striking the chamber walls (*k_wl_*), and charge lost when electrons collide with the collecting rod before depositing all their energy in the air (*k_cl_*). ICRU Report 90 suggests including the remaining correction factors, *k_ii_* and *k_w_*, in air‐kerma calculations, especially for low‐energy applications such as this project.^4^ These factors correct the measured charge for collected ions that were created by the initial, incident photon (*k_ii_*) and correct the value of ${\left(\frac{\bar{W}}{e}\right)}_{air}$ to be greater than 33.97 J/C for low‐energy secondary electrons (*k_w_*).

These correction factors are measured or calculated using Monte Carlo (MC) codes.^5^ Originally, the Attix FAC correction factors were only estimated for specific beams. For this work, we sought to calculate new correction factors using modern MC codes for monoenergetic photon beams between 5 and 50 keV. Correction factors for an arbitrary polyenergetic beam can subsequently be determined by applying a weighted average to the monenergetic correction factors based on that beam's spectrum.

## 2 MATERIALS AND METHODS

**FIGURE 2:**
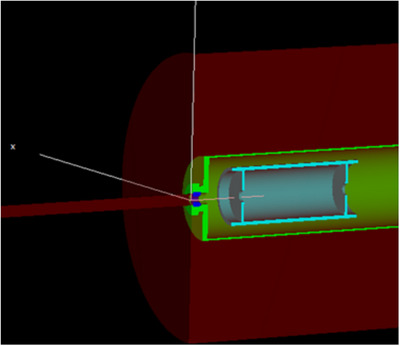
A cross‐section rendering of the model of the Attix FAC in simulations. A stainless‐steel shell (green) encloses the entire chamber. The beam passes through the beam‐defining brass aperture (dark blue) into the cylindrical aluminum collecting volume (light blue). The collecting rod was not modeled for these simulations. FAC, free‐air ionization chamber.

The MC code EGSnrc (2021 version, released 17 April, 2021) was chosen for use in this project.^6^, ^7^ As the maximum photon energy of any simulation was 50 keV, we required an MC code that would accurately model low‐energy photon‐electron transport. EGSnrc is generally regarded as the gold standard for these tasks.^8^ The EGSnrc user code, egs_fac, was employed to calculate most of the correction factors.^9^ A simplified version of the Attix FAC was modeled in the simulation. An image of the simulation geometry is shown in Figure 2. Material information for alloys used in the simulations can be found in Table 1. By defining which regions comprise the chamber volume and aperture, the code computed the aperture transmission, scatter, and wall electron loss correction factors.

**TABLE 1:** Material composition data for alloys used in the simulations.

Material	Elemental composition by weight (%)	Density (g/cm^3^)
Steel shell	Fe (99.51), Mn (0.3), C (0.1), S (0.05), P (0.04)	7.872
Steel collecting rod	Fe (70.995), Cr (18), Ni (8), Mn (2), Si (0.75), N (0.1), C (0.08), P (0.045), S (0.03)	8.030
Brass aperture	Cu (63), Zn (37)	8.473

The simulations used an egs_collimated_source with a circular shape of radius 1 cm, coaxial with the FAC, and 100 cm from the aperture. The small radius was used to avoid wasted histories where a photon interacts with the steel shell. Only a negligible fraction of photons with energies below 50 keV are able to penetrate the steel shell, making it unnecessary to simulate the entire broad beam geometry. Many default options were used for transport parameters, including the “xcom” photon cross‐sections. The minimum possible Global PCUT of 1 keV and minimum possible Global ECUT of 512 keV were assigned. Rayleigh scattering was turned on. 1E9 initial photons were simulated in parallel runs that took approximately 44 CPU hours.

A different user code, egs_chamber, and process were necessary to calculate the collecting rod electron loss correction factors.^10^ Using the same simulation geometry as the egs_fac simulations, the dose to air for the central region of the collecting volume was calculated. The simulations were then repeated with the addition of the collecting rod to the Attix FAC model. The ratio of the dose to air without the collecting rod to the dose to air with the collecting rod was defined as the collecting rod electron loss correction factor. Simulation parameters and other options were kept the same as the egs_fac simulations, except for the number of initial histories, which was raised to 1E10 photons. This resulted in a computation time of approximately 492 CPU hours per energy.

The authors chose to experimentally determine the attenuation correction factors for each beam, instead of using MC calculations. The Attix FAC is mounted on a rail system that makes it simple to shift the chamber such that the center of the collecting volume is one meter from the source, instead of the POM. The ratio of the measured ionization current in both chamber locations, corrected for the difference in fluence from the inverse‐square law, is the attenuation correction factor for whichever beam is being measured. The product of *k_ii_* and *k_w_* for the energies in this project were determined using linear approximation between the data supplied by ICRU Report 90.^4^

Beam‐specific correction factors were determined for four medium‐filtered x‐ray beams at the University of Wisconsin Accredited Dosimetry Laboratory (UWADCL): UW20‐M, UW30‐M, UW40‐M, and UW50‐M. These beams were generated using a tungsten anode and aluminum filters of various thicknesses, and the number in the beam's name indicates the tube potential in kV. The following formula was used to compute the polyenergetic correction factor for a specific beam:

(2) $$\bar{k}=\frac{\int_{E_{min}}^{E_{max}}k\left(E\right)\;\Psi \left(E\right)\;\frac{\mu_{en}}{\rho }{\left(E\right)}_{air}\;dE}{\int_{E_{min}}^{E_{max}}\Psi \left(E\right)\;\frac{\mu_{en}}{\rho }{\left(E\right)}_{air\;}dE}$$

where *k*(*E*) is the correction factor, *Ψ*(*E*) is the total (including primary and scattered) photon energy fluence, and $\frac{\mu_{en}}{\rho }{\left(E\right)}_{air}$ is the mass energy‐absorption coefficient of air for energy *E*.^11^ Mass energy‐absorption coefficients and mass energy‐transfer coefficients can be used interchangeably in the formula, as the average energy of secondary electrons lost to radiative processes in air is negligible below 50 keV.^12^ Energy fluence spectra for the UW beams were gathered from existing data, measured by Coletti.^5^ Uncertainty of the spectra is on the order of ± 4% (*k* = 2). The beam‐specific correction factors were then compared to factors estimated by Coletti. *k_ii_* and *k_w_* have not been included in this comparison, as these correction factors were not part of the standard protocol at the time of the currently accepted values.

## 3 RESULTS

**FIGURE 3:**
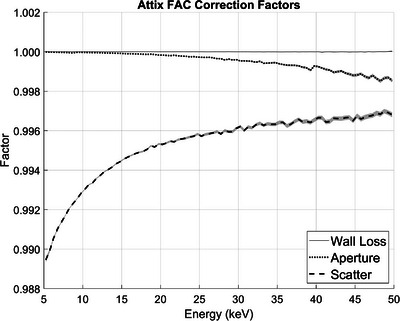
Plot of the Attix FAC correction factors as a function of incoming x‐ray photon energy. The shaded regions represent statistical uncertainties from the MC simulations. FAC, free‐air ionization chamber; MC, Monte Carlo.

Figure 3 is a plot of the aperture transmission, scatter, and wall electron loss correction factors as a function of energy for energies between 5.25 and 49.75 keV, in 0.50 keV increments. A negligible number of photons with energies below 5 keV reach the POM one meter from the source.^5^

**FIGURE 4:**
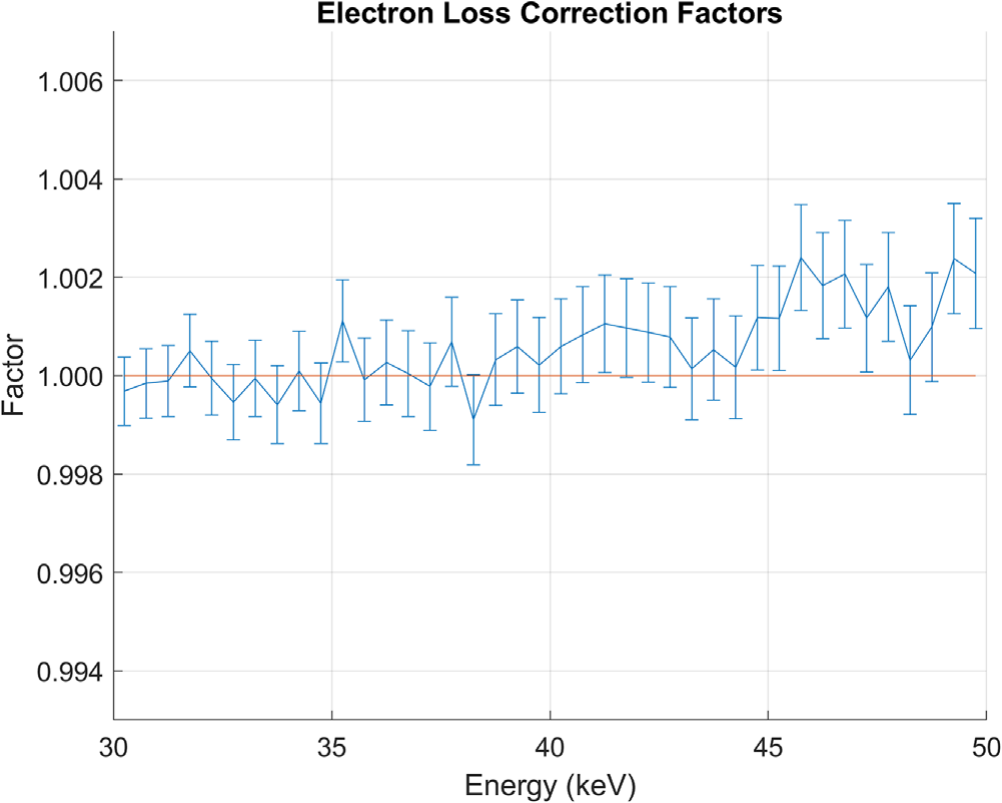
Plot of the Attix FAC collecting rod electron loss correction factors vs. photon energy. The error bars represent statistical uncertainties from the MC simulations. MC, Monte Carlo.

Figure 4 shows a plot of the collecting rod electron loss correction factors for energies between 30.25 and 49.75, in 0.50 keV increments. The egs_chamber simulations performed to determine the correction factors were significantly more time‐consuming than the egs_fac simulations. Less source particles were simulated to reduce this time burden. However, that led to increased simulation uncertainty. The collecting rod electron loss correction factors for energies below 30 keV were assumed to be unity. The collecting rod is approximately 2.225 cm from the central axis, which is further than the CSDA range of a 30 keV electron in air.^12^

Beam‐specific correction factors for the four beams are listed in Table 2. The product of the four correction factors was then compared to values determined by Coletti et al., which can be found in Table 3.^2^

**TABLE 2:** Beam‐specific correction factors for the UW‐M series beams.

Beam	*k_ap_*	*k_s_*	*k_wl_*	*k_cl_*
UW20‐M	0.9999	0.9932	1.0000	1.0000
UW30‐M	0.9999	0.9942	1.0000	1.0000
UW40‐M	0.9998	0.9947	1.0000	1.0000
UW50‐M	0.9998	0.9948	1.0000	1.0000

**TABLE 3:** The product of the four beam‐specific correction factors from Table 2 compared to values from Coletti et al. for UW‐M series beams.

Beam	Coletti et al.**2**	Stasko and culberson
UW20‐M	0.9930	0.9932
UW30‐M	0.9940	0.9941
UW40‐M	0.9945	0.9945
UW50‐M	0.9950	0.9946

Beam‐specific values of the product of *k_ii_* and *k_w_*, as well as the resulting total beam‐specific correction factors are included in Table 4.

**TABLE 4:** Beam‐specific values of *k_ii_k_w_* and total correction factors for the UW‐M series beams.

Beam	*k_ii_kw*	Total beam‐specific correction factors
UW20‐M	0.9962	0.9894
UW30‐M	0.9968	0.9909
UW40‐M	0.9972	0.9918
UW50‐M	0.9973	0.9919

**TABLE 5:** Uncertainties for calculation of beam‐specific correction factors, i.e., one of *k_ap_*, *k_s_*, *k_wl_*, or *k_cl_*.

Parameter	Type A (%)	Type B (%)
MC statistical uncertainty	0.010	
Geometric uncertainty	0.010	
Spectral uncertainty		4E‐4
Mass energy‐absorption coefficient uncertainty		0.020
A and B quadratic sum	0.014	0.020
Combined uncertainty	0.024% (*k* = 1)	
	0.048% (*k* = 2)	

### 3.1 Uncertainty analysis

Determining the uncertainty of the beam‐specific correction factors computed in this study is difficult, as there are several sources of uncertainty. These include simulation uncertainties from the FAC geometry, statistical uncertainty from the simulations, uncertainty from the measured spectra, and uncertainty in the mass energy‐absorption coefficients used in the simulations and averaging calculations. All of these uncertainties are energy‐dependent. The values listed in Table 5 reflect the greatest uncertainty in determining one of the four types of polyenergetic correction factors (i.e., *k_ap_*, *k_s_*, *k_wl_*, *k_cl_*) for each beam.

MC statistical uncertainty is a natural result of using random numbers and probabilities to determine the likely outcome of a given situation. The simulation uncertainty in each monoenergetic correction factor was weighted by the contribution of photons of that energy towards the total air kerma, so the resulting polyenergetic simulation uncertainty is an order of magnitude smaller than the monoenergetic simulation uncertainty. This makes sense, as changing one individual monoenergetic correction factor does not greatly affect the polyenergetic correction factor. The geometric uncertainty was determined by changing the dimensions of the FAC in simulation, based on the uncertainty of physical measurements of FAC components. The value given is the maximum percent change of any correction factor after modifying simulation dimensions. A spectral uncertainty of 2% was provided in Coletti's work.^5^ This value was then propagated through the calculations of the polyenergetic correction factors, and the largest uncertainty is listed in the table. As the correction factors are the weighted average of many monoenergetic factors, uncertainty in the fluence of any one specific photon energy does not result in a significant change in the final correction factor. Hence, the uncertainty in the polyenergetic correction factors is four orders of magnitude smaller than the uncertainty in the spectra.

Finally, estimating uncertainties of mass energy‐absorption coefficients is challenging, as there are several datasets that provide different values, especially at the low energies that were used in this work.^4^ The best estimate of the uncertainty in determining the mass energy‐absorption coefficient for a given energy is approximately 2.5% below 50 keV.^13^ In order to estimate the effect of the uncertainty of the mass‐energy absorption coefficient on the uncertainty of computing polyenergetic correction factors, some monoenergetic correction factor simulations were repeated using the “mcdf‐xcom” cross‐sections option, which uses renormalized photoelectric effect cross‐sections computed with Pratt's normalization screening approximation. This method helps account for electron correlations that are neglected in the conventional independent‐electron model of the photoelectric effect.^14^ Values for energies that were not simulated again were derived through linear interpolation. The four polyenergetic correction factors for each beam were then recalculated, and the largest percent difference between the original values and the new values is given as an estimate of the uncertainty of the mass‐energy absorption coefficients in Table 4.

## 4 DISCUSSION

The trends observed in the correction factor plots matched expectations. The aperture transmission correction factor became more significant as photon energy increased, as higher energy photons were more likely to pass through or scatter off the beam aperture without being attenuated. The scatter correction factor was more significant at lower photon energies. The wall electron loss correction factor was negligible for all energies. The Attix FAC was constructed such that the walls of the chamber would not interfere with the 50 keV electrons leaving the central collection volume.

However, the collecting rod electron loss factor had previously been assumed to be negligible as well for all energies. While that is true for lower energies, there does appear to be some higher energy secondary electrons that may collide with the collecting rod. The number of higher energy photons (>40 keV) within any beam measured with this chamber is likely to be low, so overall, this correction remained negligible for the investigated beams.

The correction factors estimated by Coletti et al. and our correction factors agreed within approximately 0.04% for all beams investigated. These small differences are likely the result of two factors: advances and improvements in MC physics simulations and simulation uncertainty in our work.

Each polyenergetic correction factor other than the scatter correction factor is within the MC simulation uncertainty of unity, so the only correction factor that has a significant role in the total beam‐specific correction factors is the scatter correction. It is also important to recognize that the *k_ii_* and *k_w_* corrections significantly affect the total beam‐specific correction, if they are included in the analysis. Moving forward, these corrections will be part of air‐kerma measurements with this FAC.

It is important to note that the uncertainty analysis in this work only considers the uncertainties that affect the calculated correction factors. When measuring air‐kerma rates with an FAC, there are uncertainties that arise from the measured current, the density of air, and other components. The final uncertainty for those measurements will be significantly larger than the correction factor uncertainties presented in this study.

## 5 CONCLUSIONS

Monoenergetic correction factors for air‐kerma measurements with the Attix FAC at the UWMRRC were determined through the use of the EGSnrc user codes, egs_fac and egs_chamber. Beam‐specific correction factors were calculated based on the beams’ photon fluence spectra and were compared to accepted values. Agreement within 0.1% was found for the four beam‐specific total correction factors investigated. The process for determining beam‐specific correction factors carried out in this work will be repeated for other low‐energy x‐ray beams at the UWMRRC. This will allow us to characterize the air‐kerma rates of several beams that have yet to be measured. Other institutions could also determine correction factors for their own FACs and photon beams following a similar method.

## CONFLICT OF INTEREST STATEMENT

The authors declare no conflicts of interest.

## References

[1] Burns DT , Büermann L . Free‐air ionization chambers. Metrologia. 2009;46(2):S9‐S23. doi:10.1088/0026-1394/46/2/S02

[2] Coletti JG , Pearson DW , DeWerd LA . Mammography exposure standard: design and characterization of free‐air ionization chamber. Rev Sci Instrum. 1995;66(3):2574‐2577. doi:10.1063/1.1145590

[3] King EJ , Viscariello NN , DeWerd LA . Development of standard x‐ray beams for calibration of radiobiology cabinet and conformal irradiators. Radiat Res. 2021;197(2). doi:10.1667/RADE-21-00121.1 34634111

[4] Seltzer S , Fernández‐Varea J , Andreo P , et al. Key data for ionizing‐radiation dosimetry: measurement standards and applications. ICRU Report 90. J ICRU. 2016;14:1‐110.

[5] Coletti JG . Effects of Calibration Spectra on Mammographic Exposure Measurement. Dissertation. 1995. https://medphysics.wisc.edu/files/thesis/coletti_john_95.pdf

[6] Kawrakow I . Accurate condensed history Monte Carlo simulation of electron transport. I. egs nrc, the new egs4 version. Med Phys. 2000;27(3):485‐498. doi:10.1118/1.598917 10757601

[7] Kawrakow I , Rogers DWO , Mainegra‐Hing E , Tessier F , Townson R , Walters B . EGSnrc toolkit for Monte Carlo simulation of ionizing radiation transport. Published online 2000. doi:10.4224/40001303

[8] Rogers DWO . Low energy electron transport with EGS. Nuclear Instruments and Methods in Physics Research Section A: Accelerators, Spectrometers, Detectors and Associated Equipment. Elsevier; 1984:535‐548. doi:10.1016/0168-9002(84)90213-4

[9] Mainegra‐Hing E , Reynaert N , Kawrakow I . Novel approach for the Monte Carlo calculation of free‐air chamber correction factors: novel approach for the MC calculation of FAC corrections. Med Phys. 2008;35(8):3650‐3660. doi:10.1118/1.2955551 18777925

[10] Wulff J , Zink K , Kawrakow I . Efficiency improvements for ion chamber calculations in high energy photon beams. Med Phys. 2008;35(4):1328‐1336. doi:10.1118/1.2874554 18491527

[11] Büermann L . The PTB Free‐Air Ionization Chambers. Physikalisch‐Technische Bundesanstalt (PTB); 2022:3235302 bytes. doi:10.7795/120.20220324

[12] Seltzer S . Stopping‐powers and range tables for electrons, protons, and helium ions, NIST standard reference database 124 124 [Dataset]. National Institute of Standards and Technology. 1993. doi:10.18434/T4NC7P

[13] Andreo P , Burns DT , Salvat F . On the uncertainties of photon mass energy‐absorption coefficients and their ratios for radiation dosimetry. Phys Med Biol. 2012;57(8):2117‐2136. doi:10.1088/0031-9155/57/8/2117 22451262

[14] Sabbatucci L , Salvat F . Theory and calculation of the atomic photoeffect. Radiat Phys Chem. 2016;121:122‐140. doi:10.1016/j.radphyschem.2015.10.021

